# Live and Heat-Killed *Lactiplantibacillus plantarum* HY7715 Attenuate D-Galactose-Induced Aging-Related Brain Changes and Blood-Brain Barrier Disruption with Gut Microbiota Modulation in Mice

**DOI:** 10.4014/jmb.2609.09057

**Published:** 2026-10-21

**Authors:** Jimin Baik, Hyeonjun Gwon, Joo-Yun Kim, Jae-Jung Shim, Jae-Hwan Lee

**Affiliations:** R&BD Center, hy Co., Ltd., 22, Giheungdanji-ro 24beon-gil, Giheung-gu, Yongin-si 17086, Gyeonggi-do, Republic of Korea

**Keywords:** *Lactiplantibacillus plantarum* HY7715, D-galactose-induced accelerated aging, Blood-brain barrier, Gut microbiota, Intestinal barrier, Gut-brain axis

## Abstract

Aging is associated with changes in systemic homeostasis, intestinal barrier integrity, and brain function. This study investigated the effects of *Lactiplantibacillus plantarum* HY7715 on aging-related alterations in a D-galactose-induced accelerated aging mouse model. Male C57BL/6J mice were administered D-galactose for 8 weeks and orally treated with live or heat-killed HY7715. D-galactose increased circulating advanced glycation end products (AGEs), blood-brain barrier (BBB) permeability, hippocampal inflammation, and aging-related molecular changes, while impairing cortical and colonic tight junction-associated markers. Both live and heat-killed HY7715 reduced serum AGE levels, Evans blue leakage, and hippocampal inflammatory responses, while restoring CLDN5 immunoreactivity, tight junction-associated gene expression, and antioxidant-related gene expression. HY7715 also attenuated senescence- and apoptosis-related changes in the hippocampus and colonic tight junction- and inflammation-related gene expression changes. Gut microbiota analysis showed taxon-specific changes following HY7715 treatment, including significant increases in *Bifidobacterium* in both HY7715-treated groups, whereas live HY7715 was associated with significant changes in microbial community structure. Several microbial taxa were correlated with serum AGEs, BBB permeability, and neuroinflammation-related parameters. Together, these results show that both live and heat-killed HY7715 attenuated multiple aging-related changes in the D-galactose-induced accelerated aging model, accompanied by changes in gut microbiota composition.

## Introduction

Brain aging is a complex biological process characterized by progressive structural and functional decline and is a major risk factor for neurodegenerative disorders [1, 2]. Persistent oxidative stress, chronic low-grade inflammation, mitochondrial dysfunction, and cellular senescence progressively impair neuronal homeostasis during aging [2, 3]. These processes contribute to inflammaging and increase the vulnerability of the aging brain to functional decline [3]. The hippocampus and cerebral cortex are particularly vulnerable to aging-related stress because of their central roles in memory and cognition [2].

Aging also affects peripheral systems, including the intestine, and is accompanied by changes in the gut microbiota [4, 5]. Natural and experimentally induced aging can alter intestinal barrier function, immune responses, and microbial composition [4, 6]. These changes may contribute to systemic inflammation and metabolic imbalance through microbial products, inflammatory mediators, and microbiota-derived metabolites [4, 5]. The gut-brain axis therefore provides a framework for understanding how intestinal and microbial changes may accompany brain aging [5, 7].

The blood-brain barrier (BBB) maintains the tightly regulated microenvironment required for normal brain function [8]. BBB integrity depends largely on specialized brain endothelial cells and tight junction proteins, including claudin-5, occludin, and ZO-1 [8, 9]. Aging-related oxidative, inflammatory, and glycation-associated stress can impair these components, increasing barrier permeability and exposure of the brain to circulating factors [8, 9]. Therefore, BBB dysfunction may represent an important connection between systemic aging-related stress and central neuroinflammatory responses.

Chronic D-galactose exposure is widely used as an accelerated aging model because it reproduces several aging-related features within a relatively short period [10, 11]. Excessive D-galactose induces oxidative stress, inflammation, mitochondrial dysfunction, metabolic dysregulation, and senescence-like phenotypes in multiple tissues [10, 11]. It also promotes non-enzymatic glycation and accumulation of advanced glycation end products (AGEs), which are associated with inflammatory and oxidative stress-related signaling [12]. In the brain, D-galactose-induced accelerated aging has been associated with senescence-related molecular changes, neuroinflammation, apoptosis, impaired antioxidant defense, reduced tight junction integrity, and increased BBB permeability [11, 13, 14]. However, the relationships among gut microbial alterations, intestinal barrier-related responses, systemic AGE accumulation, and BBB dysfunction remain incompletely understood.

Among potential interventions, *Lactiplantibacillus plantarum* strains have been investigated for their effects on immune regulation, barrier function, and host metabolism [15, 16]. The kimchi-derived strain *Lactiplantibacillus plantarum* HY7715 has shown beneficial effects in several experimental models [17, 18]. HY7715 ameliorated age-related sarcopenic changes while improving skeletal muscle mass, enhancing mitochondrial biogenesis, and restoring gut microbiota composition [18]. It also attenuated oxidative stress, apoptosis, and neuroinflammatory responses in the hippocampus in a restraint stress model [19]. Previous studies further suggest that HY7715 can influence both host responses and the gut microbial environment [18, 20].

Whether live and heat-killed HY7715 can attenuate D-galactose-induced AGE accumulation, BBB disruption, and brain-related alterations in an accelerated aging model has not been established. Therefore, the present study evaluated the effects of live and heat-killed HY7715 on serum AGE levels, BBB permeability, cortical tight junction-associated markers, hippocampal neuroinflammatory, senescence-, antioxidant-, and apoptosis-related responses, colonic barrier-related gene expression, and gut microbiota composition in D-galactose-induced accelerated aging mice. Correlations between microbial taxa and aging-related phenotypic markers were also examined to explore associations between gut microbiota composition and systemic and brain-related responses.

## Materials and Methods

### Bacterial Strain Preparation

*Lactiplantibacillus plantarum* HY7715, originally isolated from kimchi, was maintained in the probiotic strain library of hy Co., Ltd. Prior to production, the stored HY7715 stock was streaked onto de Man, Rogosa, and Sharpe (MRS) agar, and an isolated colony was cultured in MRS broth. The identity of the strain was confirmed by 16S rRNA gene sequencing performed by Macrogen, Inc. (Republic of Korea). Following strain confirmation, live and heat-killed HY7715 powders used in the animal experiment were manufactured at the Pyeongtaek Probiotics Plant of hy Co., Ltd. (Republic of Korea). Prior to animal administration, the viable cell count of the live HY7715 powder was determined by serial dilution and plating on MRS agar. The dose of the heat-killed preparation was calculated based on the viable cell count determined before heat inactivation during production.

### Animal Study Design

Male C57BL/6J mice, 7 weeks old, were purchased from DooYeol Biotech Co., Ltd. (Republic of Korea). The mice were housed under standard specific-pathogen-free (SPF) conditions (22 ± 2°C and 50 ± 10% humidity, and a 12 h light/dark cycle) with ad libitum access to standard chow and water. All experimental procedures were approved by the Institutional Animal Care and Use Committee (IACUC) of hy Co., Ltd. Approval number AEC-2026-0005-Y. After 1 week of acclimation, the mice were randomly assigned to four experimental groups with 10 mice per group. The live HY7715 and heat-killed HY7715 groups were designated as 7715L and 7715K, respectively. The NOR group served as the normal control and received vehicle treatment. The CON group received D-galactose at 150 mg/kg/day. The 7715L group received D-galactose at 150 mg/kg/day and live HY7715 at 1 × 10^9^ CFU/kg/day. The 7715K group received D-galactose at 150 mg/kg/day and heat-killed HY7715 equivalent to 1 × 10^9^ CFU/kg/day before heat killing. D-galactose was administered subcutaneously once daily for 8 weeks to the CON, 7715L, and 7715K groups. The NOR group received an equivalent volume of saline by the same route. Live or heat-killed HY7715 suspended in PBS was orally administered once daily for 8 weeks to the 7715L and 7715K groups, respectively. The NOR and CON groups received an equivalent volume of PBS by oral administration during the same period. Within each group, seven mice were used for biochemical, molecular, and immunofluorescence analyses, whereas the remaining three mice were used for the Evans blue extravasation assay. For the seven mice used for molecular and immunofluorescence analyses, the brain was bisected along the midline. One hemisphere was fixed for immunofluorescence analysis, whereas the cerebral cortex and hippocampus were dissected from the contralateral hemisphere, snap-frozen, and stored at -80°C until RNA extraction.

### Measurement of Serum AGEs

Serum levels of advanced glycation end products (AGEs) were measured using the OxiSelect^TM^ Advanced Glycation End Product (AGE) Competitive ELISA Kit (Cell Biolabs, USA; catalog no. STA-817) according to the manufacturer's instructions. Briefly, 50 μL of each serum sample or AGE-BSA standard was added to AGE-conjugate-coated wells and incubated with diluted anti-AGE antibody for 1 h at room temperature. After washing, the wells were incubated with diluted HRP-conjugated secondary antibody for 1 h at room temperature. Then, 100 μL of substrate solution was added to each well and incubated at room temperature for 2–30 min. The reaction was stopped by adding 100 μL of stop solution, and absorbance was measured at 450 nm using a microplate reader. AGE levels were calculated from a standard curve generated using the AGE-BSA standards and expressed as μg/mL AGE-BSA equivalents.

### Evaluation of Blood-Brain Barrier (BBB) Permeability

Blood-brain barrier permeability was assessed using the Evans blue extravasation assay. Mice were anesthetized with isoflurane and injected retro-orbitally with 50 μL of 2% Evans blue solution. The dye was allowed to circulate for 1 hour. The mice were then anesthetized and perfused with PBS to remove residual Evans blue from the circulation. The brains were rapidly dissected and weighed. To extract the extravasated Evans blue dye, the brain tissues were cut into small pieces and incubated in 1 mL of formamide at 65°C for 24 h. The extracts were then centrifuged at 14,000 × g for 20 min at 4°C. The absorbance of the filtered supernatants was measured at 620 nm using a microplate reader, and absorbance at 740 nm was subtracted for background correction. The corrected absorbance values (OD620 – OD740) were normalized to the corresponding brain tissue weight and expressed as OD/g tissue.

### Brain Immunostaining and Image Analysis

Mice were euthanized, and the brains were carefully isolated and fixed in 4% paraformaldehyde (PFA) at 4°C for 24 h. After fixation, the brains were rinsed with PBS and incubated in 30% sucrose in PBS at 4°C for cryoprotection until the tissues sank. The brains were then embedded in Tissue-Tek O.C.T. compound (Sakura Finetek, Japan; catalog no. 4583) and rapidly frozen. Brain sections were prepared at a thickness of 10 μm using a cryostat (Leica Biosystems, Germany; catalog no. CM1950), and the sections were mounted onto SuperFrost Plus microscope slides (Thermo Fisher Scientific, USA; catalog no. J1800AMNZ). To block nonspecific binding, the sections were incubated for 1 h at room temperature in blocking solution containing 0.3% Triton X-100 and 5% BSA in PBS. After blocking, the sections were incubated with primary antibodies diluted in staining buffer of the same composition. The following primary antibodies were used: rabbit anti-Claudin-5 antibody (1:1,000; Invitrogen, USA, catalog no. 34−1600) and rabbit anti-Iba1 antibody (1:1,000; Abcam, UK, catalog no. ab178846). Primary antibody incubation was performed overnight at 4°C. After washing with PBS, the sections were incubated with AffiniPure goat anti-rabbit IgG H+L, Alexa Fluor 488 antibody (1:2,000, Jackson ImmunoResearch, USA, catalog no. 111-545-144) for 2 h at room temperature. The sections were then rinsed with PBS and counterstained with DAPI (1:5,000; Invitrogen, catalog no. D3571) for nuclear visualization. After additional washing with PBS, the sections were mounted using Fluoromount aqueous mounting medium (Sigma-Aldrich, USA, catalog no. F4680) and stored in the dark at 4°C. Fluorescence images were acquired using an LSM 800 confocal microscope (ZEISS, Germany) at 200× magnification under identical imaging settings for all samples. Z-stack images were acquired, and maximum-intensity projection images were generated using ZEN software (version 3.10; ZEISS). For each marker, one brain section per animal was analyzed. For CLDN5 analysis, a single 200× field of view encompassing the cerebral cortex was acquired, whereas for Iba1 analysis, a single 200× field of view encompassing the hippocampal CA1 region was acquired from each animal. The entire field of view was used as the region of interest (ROI). CLDN5-positive area and fluorescence intensity, as well as Iba1-positive cell number and soma area, were quantified using Fiji/ImageJ software (National Institutes of Health, USA). Thus, one image-derived value per animal was used for statistical analysis.

### Total RNA Isolation and Quantitative Real-Time PCR (RT-qPCR)

Total RNA was extracted from the cerebral cortex, hippocampus, and colon using the Easy-spin Total RNA Extraction Kit (iNtRON Biotechnology, Republic of Korea) according to the manufacturer's instructions. The isolated RNA was reverse-transcribed into complementary DNA using the Omniscript Reverse Transcription Kit (Qiagen, Germany). Quantitative real-time PCR was performed on a QuantStudio 6 Real-Time PCR System (Applied Biosystems, USA) using TaqMan^TM^ Gene Expression Assays (Applied Biosystems). Tight junction-associated genes were analyzed in the cerebral cortex; inflammation-, senescence-, antioxidant-, and apoptosis-related genes were analyzed in the hippocampus; and tight junction- and inflammation-related genes were analyzed in the colon. The target genes and corresponding TaqMan Gene Expression Assay IDs are listed in Table 1. Relative gene expression was calculated using the 2^-ΔΔCt^ method and normalized to glyceraldehyde-3-phosphate dehydrogenase (Gapdh; Mm99999915_g1). The NOR group was used as the calibrator for relative gene expression analysis. Samples for which no Ct value was obtained within 40 amplification cycles were considered below the detection limit.

### Fecal Microbiota Sequencing and Bioinformatics

Fecal pellets were collected from each mouse and used for 16S rRNA amplicon sequencing by Macrogen. Genomic DNA was extracted from fecal samples using the QIAamp Fast DNA Stool Mini Kit (Qiagen) according to the manufacturer’s instructions. The V3-V4 hypervariable region of the bacterial 16S rRNA gene was amplified using the 341F and 806R primer pair. Amplicon libraries were sequenced using an Illumina MiSeq i100 platform (Illumina, USA). Raw sequencing data were processed using QIIME 2 (version 2024.5). Paired-end reads were quality-filtered, denoised, merged, and chimera-filtered using the DADA2 plugin to generate amplicon sequence variants (ASVs). Taxonomic classification was performed using the SILVA 138 full-length reference database. Alpha diversity was evaluated using Faith’s phylogenetic diversity and observed features, and differences among groups were analyzed using the Kruskal-Wallis test. Beta diversity was evaluated using unweighted and weighted UniFrac distances and visualized by principal coordinate analysis (PCoA). Differences in beta diversity among groups were assessed by PERMANOVA. Relative taxonomic abundance was calculated to compare fecal microbial composition among experimental groups. For visualization of low-abundance taxa, *Faecalibaculum* was excluded from selected taxonomic plots, and the remaining taxa were re-normalized to 100%. Taxa with a relative abundance of 1% or higher in at least one group were classified as major taxa, whereas taxa below 1% were grouped as Minor. All statistical analyses of taxonomic differences were performed using the original taxonomic dataset before exclusion of *Faecalibaculum*. Differentially abundant taxa were identified using LEfSe with an LDA score cutoff of 2.0, and the default significance threshold of *p* < 0.05 was applied. For selected taxa, putative species-level annotation was performed by BLASTn search against the NCBI nucleotide database. Species-level assignments were considered exploratory and were not used as definitive taxonomic identification. The raw 16S rRNA gene sequencing data have been deposited in the NCBI Sequence Read Archive under BioProject accession number PRJNA1533068.

### Correlation Analysis

Spearman correlation analysis was performed to assess associations between differentially abundant fecal bacterial taxa and aging-related phenotypic markers, including serum AGE levels, brain Evans blue extravasation, cortical Claudin-5 immunofluorescence parameters, and hippocampal Iba1 immunofluorescence parameters. Correlation matrices were presented as heatmaps, and significant correlations were indicated by asterisks. Statistical significance was set at *p* < 0.05.

### Statistical Analysis

Statistical analyses were performed using GraphPad Prism version 10 (GraphPad Software, USA). Data are presented as the mean ± standard deviation, SD. For comparisons among three or more groups, data were analyzed using one-way ANOVA followed by Dunnett’s multiple comparisons test. Selected comparisons were performed using NOR or CON as the reference group, as indicated in each figure. Changes in body weight over time were analyzed using two-way ANOVA followed by Dunnett’s multiple comparisons test. Regarding the bioinformatic datasets, discrepancies in alpha diversity were statistically appraised using the Kruskal-Wallis non-parametric test, while divergences in overall beta diversity structures were confirmed via Permutational Multivariate Analysis of Variance (PERMANOVA). Statistical significance was set at *p* < 0.05.

## Results

### Effects of HY7715 on Body Weight and Serum AGE Levels in D-Galactose-Induced Accelerated Aging Mice

Body weight progressively increased throughout the 8-week experimental period in all groups. Compared with the NOR group, the CON group showed a relatively lower increase in body weight during the later weeks of D-galactose administration. In contrast, both the 7715L and 7715K groups showed greater body weight increases than the CON group during the latter part of the experimental period (Fig. 1B). Serum AGE concentrations increased from 5.26 ± 2.30 μg/mL in the NOR group to 17.33 ± 0.41 μg/mL in the CON group (*p* < 0.001). Both the 7715L and 7715K groups showed significantly lower serum AGE concentrations than the CON group, reaching 6.71 ± 3.91 μg/mL and 6.12 ± 1.60 μg/mL, respectively (*p* < 0.001; Fig. 1C).

### HY7715 Reduces Blood-Brain Barrier Permeability in D-Galactose-Induced Accelerated Aging Mice

Representative images showed greater Evans blue accumulation in the CON group than in the NOR group, whereas lower accumulation was observed in the 7715L and 7715K groups (Fig. 2A). Quantitative analysis showed that Evans blue extravasation was significantly increased from 0.05 ± 0.004 OD/g in the NOR group to 0.39 ± 0.007 OD/g in the CON group (*p* < 0.001). Both the 7715L and 7715K groups showed significantly lower Evans blue extravasation than the CON group, reaching 0.08 ± 0.002 OD/g and 0.07 ± 0.004 OD/g, respectively (*p* < 0.001; Fig. 2B).

### HY7715 Preserves Cortical Tight Junction-Associated Markers in D-Galactose-Induced Accelerated Aging Mice

To further investigate the effects of HY7715 on BBB integrity, the expression of the tight junction protein CLDN5 was examined in the cerebral cortex by immunofluorescence, and the mRNA expression of tight junction-associated genes was evaluated by RT-qPCR after 8 weeks of D-galactose administration. Representative images showed lower CLDN5 staining in the cerebral cortex of the CON group compared with the NOR group, whereas greater CLDN5 staining was observed in the 7715L and 7715K groups (Fig. 3A). Quantitative analysis showed that the CLDN5-positive area decreased from 1.75 ± 0.49 % in the NOR group to 1.03 ± 0.42 % in the CON group (*p* < 0.05). The CLDN5-positive area increased to 1.70 ± 0.58 % and 2.06 ± 0.29 % in the 7715L and 7715K groups, respectively (vs. CON, *p* < 0.05 and *p* < 0.001; Fig. 3B). Relative CLDN5 fluorescence intensity was also significantly lower in the CON group than in the NOR group (*p* < 0.01), whereas both the 7715L and 7715K groups showed significantly higher fluorescence intensity than the CON group (*p* < 0.05; Fig. 3C). Cortical expression of *Cldn5*, *Tjp1*, and *Ocln* was reduced in the CON group to 0.82 ± 0.05-, 0.84 ± 0.04-, and 0.86 ± 0.06-fold relative to NOR, respectively (*p* < 0.001 for all genes). Compared with the CON group, *Cldn5* expression was significantly increased in both 7715L and 7715K groups (*p* < 0.001; Fig. 3D). Similarly, *Tjp1* expression was significantly increased in the 7715L and 7715K groups (*p* < 0.001 and *p* < 0.01, respectively; Fig. 3E), as was *Ocln* expression (*p* < 0.01 and *p* < 0.05, respectively; Fig. 3F).

### HY7715 Attenuates Hippocampal Microglial Reactivity and Inflammatory Gene Expression

To investigate the effects of HY7715 on neuroinflammatory responses associated with D-galactose-induced accelerated aging, microglial reactivity was evaluated based on Iba1-positive cell number and soma area in the hippocampal CA1 region, and the mRNA expression of inflammatory cytokines was evaluated by RT-qPCR after 8 weeks of D-galactose administration. The CON group showed increased Iba1 immunoreactivity in the hippocampal CA1 region compared with the NOR group, whereas lower Iba1 immunoreactivity was observed in the 7715L and 7715K groups (Fig. 4A). Quantitative analysis showed that the number of Iba1-positive cells increased from 105.13 ± 11.35 cells/mm^2^ in the NOR group to 148.81 ± 29.22 cells/mm^2^ in the CON group (*p* < 0.01). The number of Iba1-positive cells was significantly reduced to 109.99 ± 16.99 cells/mm^2^ in the 7715L group (*p* < 0.05) and 105.13 ± 24.10 cells/mm^2^ in the 7715K group (*p* < 0.01) compared with the CON group (Fig. 4B). The average soma area of Iba1-positive cells was also increased from 36.58 ± 2.10 μm^2^ in the NOR group to 42.59 ± 2.12 μm^2^ in the CON group (*p* < 0.01). Both the 7715L and 7715K groups showed significantly reduced soma areas, reaching 35.03 ± 1.29 μm^2^ in the 7715L group and 36.90 ± 2.89 μm^2^ in the 7715K group (*p* < 0.001 and *p* < 0.01, respectively; Fig. 4C). Hippocampal *Tnf-α*, *IL-1β*, and *IL-6* expression increased to 4.22 ± 0.33-, 53.16 ± 16.52-, and 22.58 ± 7.82-fold in the CON group, respectively (*p* < 0.001). Both HY7715 groups significantly reduced the expression of all three genes compared with CON (*p* < 0.001; Fig. 4D−F), with *Tnf-α* levels of 2.04 ± 0.47- and 1.87 ± 0.72-fold, *IL-1β* levels of 3.25 ± 0.67- and 2.62 ± 0.55-fold, and *IL-6* levels of 2.55 ± 0.52- and 3.09 ± 0.94-fold in the 7715L and 7715K groups, respectively.

### HY7715 Modulates Senescence-, Antioxidant-, and Apoptosis-Related Gene Expression in the Hippocampus

To investigate the effects of HY7715 on aging-associated molecular changes in the hippocampus, the mRNA expression levels of senescencerelated genes, *Trp53* (*p53*) and *Cdkn1a* (*p21*), antioxidant-related genes (*Gpx1*, *Gpx2*, *Sod1*, and *Sod2*), and apoptosis-related genes (*Bax* and *Bcl-2*), were evaluated by RT-qPCR after 8 weeks of D-galactose administration. Compared with the NOR group, the CON group showed significantly increased expression of *p53* and *p21*, reaching 1.24 ± 0.05- and 2.17 ± 0.18-fold, respectively (*p* < 0.001). Both the 7715L and 7715K groups showed significantly lower *p53* expression than the CON group (*p* < 0.001; Fig. 5A). Similarly, *p21* expression was reduced to 1.31 ± 0.07-fold and 1.21 ± 0.19-fold in the 7715L and 7715K groups, respectively (*p* < 0.001; Fig. 5B). The antioxidant-related genes *Gpx1*, *Gpx2*, *Sod1*, and *Sod2* were reduced in the CON group to 0.90 ± 0.07-, 0.64 ± 0.02-, 0.69 ± 0.12-, and 0.69 ± 0.04-fold, respectively (*p* < 0.01 or *p* < 0.001). Both HY7715 groups significantly increased the expression of these genes compared with CON (Fig. 5C–F). *Bax* expression increased to 1.85 ± 0.25-fold in the CON group compared with the NOR group (*p* < 0.001). This increase was reduced to 1.13 ± 0.07- and 1.17 ± 0.03-fold in the 7715L and 7715K groups, respectively (*p* < 0.001; Fig. 5G). Conversely, *Bcl-2* expression decreased to 0.81 ± 0.04-fold in the CON compared with the NOR (*p* < 0.001) and was significantly increased by both HY7715 treatments (*p* < 0.01; Fig. 5H).

### HY7715 Improves Colonic Tight Junction- and Inflammation-Related Gene Expression

The CON group showed reduced expression of *Tjp1* and *Ocln*, reaching 0.81 ± 0.09- and 0.82 ± 0.06-fold, respectively (*p* < 0.01 and *p* < 0.001 vs. NOR). Both 7715L and 7715K increased *Tjp1* and *Ocln* expression compared with CON (Fig. 6A and B). In contrast, *Cldn2* expression increased to 1.66 ± 0.17-fold in CON (*p* < 0.001 vs. NOR) and was reduced to 1.25 ± 0.14- and 1.23 ± 0.17-fold in the 7715L and 7715K groups, respectively (*p* < 0.001; Fig. 6C). Colonic *Tnf-α*, *IL-1β*, and *IL-6* expression increased to 3.27 ± 0.77-, 2.31 ± 0.33-, and 1.98 ± 0.16-fold in the CON group, respectively (*p* < 0.001 vs. NOR). Both HY7715 groups significantly reduced all three inflammatory genes compared with CON (*p* < 0.001; Fig. 6D–6F).

### Effects of HY7715 Supplementation on Gut Microbial Alpha Diversity and Beta Diversity

Neither Faith’s PD nor observed features differed significantly among groups based on the Kruskal-Wallis test (Fig. 7A and 7B). PERMANOVA showed no significant difference between NOR and CON for unweighted UniFrac distance (*p* = 0.066), whereas CON differed significantly from 7715L (*p* = 0.023) but not 7715K (Fig. 7C). Similarly, weighted UniFrac analysis showed no significant difference between NOR and CON (*p* = 0.052), whereas CON and 7715L differed significantly (*p* = 0.010) and CON and 7715K did not (Fig. 7D).

### Effects of HY7715 on Fecal Microbial Composition in D-Galactose-Induced Accelerated Aging Mice

In the full taxonomic profile, *Faecalibaculum* was the dominant taxon across all groups and accounted for more than 70% of the total microbial abundance (Supplementary Fig. S1). Differentially abundant taxa were identified by LEfSe analysis with an LDA score > 2.0 and are presented in Supplementary Fig. S2. In the NOR versus CON comparison, LEfSe analysis showed significantly lower relative abundances of *Faecalibaculum* and *Erysipelotrichaceae* and significantly higher relative abundances of *Desulfovibrio*, *Alistipes*, *Gemella*, and *Anaerotruncus* in the CON group. To visualize changes in lower-abundance taxa, *Faecalibaculum* was excluded, and the remaining taxa were re-normalized to 100% (Fig. 8A). In this *Faecalibaculum*-excluded taxonomic profile, the CON group showed a tendency toward decreased relative abundance of *Bifidobacterium* and *Akkermansia* compared with the NOR group, although these changes were not statistically significant. Compared with the CON group, LEfSe analysis showed that the 7715L group had significantly higher relative abundances of *Bifidobacterium* and *Lactiplantibacillus* and significantly lower relative abundances of *Desulfovibrio*, *Tuzzerella*, *Anaerotruncus*, and *Marvinbryantia*. The 7715K group showed significantly higher relative abundances of *Bifidobacterium*, *Psychrobacter*, and *Anaeroplasma* and significantly lower relative abundances of *Oscillibacter*, *Tuzzerella*, *Anaerotruncus*, and *Marvinbryantia* than the CON group. Species-level inference of selected taxa was performed using NCBI BLASTn analysis (Fig. 8B-8E). An ASV showing the highest BLASTn similarity to *Lactiplantibacillus plantarum* was observed only in the 7715L group (Fig. 8B). An ASV putatively assigned to *Desulfovibrio* sp. showed significantly higher relative abundance in the CON group than in the NOR group (*p* < 0.01). Although its relative abundance was numerically lower in the 7715L and 7715K groups than in the CON group, these treatment-related differences were not statistically significant (Fig. 8C). An ASV putatively assigned to *Bifidobacterium* sp. showed numerically lower relative abundance in the CON group and numerically higher relative abundance in the 7715L and 7715K groups than in the CON group, although these differences were not statistically significant (Fig. 8D). Notably, the relative abundance of an ASV putatively assigned to *Bifidobacterium pseudolongum* was significantly higher in the 7715L group than in the CON group (Fig. 8E).

### Correlations Between Fecal Microbial Taxa and Aging-Related Phenotypic Markers

Spearman correlation analysis identified several associations between microbial taxa and aging-related phenotypic markers (Fig. 9). *Bifidobacterium* was negatively correlated with serum AGEs and hippocampal Iba1-positive soma area and positively correlated with CLDN5-positive area. Evans blue leakage was negatively correlated with *Akkermansia* and positively correlated with *Desulfovibrio*.

## Discussion

The present study demonstrated that both live and heat-killed *L. plantarum* HY7715 attenuated systemic, cerebral, and intestinal alterations induced by chronic D-galactose administration. D-galactose increased circulating advanced glycation end products (AGEs), enhanced blood-brain barrier (BBB) permeability, reduced cortical tight junction-associated markers, and promoted hippocampal inflammation, senescence-related responses, altered antioxidant-related gene expression, and apoptosis-related changes. Changes in tight junction-associated and inflammatory gene expression were also observed in the colon. Although 7715L and 7715K produced broadly similar improvements in these host phenotypes, both treatments were also associated with changes in selected gut microbial taxa.

Chronic D-galactose administration is widely used to induce accelerated aging-related phenotypes. D-galactose is a reducing sugar capable of promoting non-enzymatic glycation and AGE formation in vivo. Prolonged exposure increases circulating AGEs and is associated with impaired antioxidant defenses, inflammation, mitochondrial dysfunction, and aging-related functional changes. Importantly, inhibition of AGE formation with aminoguanidine has been shown to attenuate several D-galactose-induced abnormalities, supporting a role for excessive glycation in this model [10, 12]. In our study, D-galactose markedly increased serum AGE concentrations and was accompanied by a lower increase in body weight during the later experimental period. Both 7715L and 7715K reduced serum AGE concentrations to levels close to those of the NOR group and showed greater body weight gain than the CON group. These results support successful induction of a glycation-associated aging phenotype and indicate that both forms of HY7715 attenuated the increase in circulating AGE levels. The underlying mechanism cannot be determined from the current data because AGE formation and clearance were not directly measured.

The increase in circulating AGEs occurred together with marked BBB dysfunction. Evans blue is largely restricted from entering brain tissue when the BBB is intact because it binds circulating albumin. Its accumulation in brain tissue therefore reflects increased BBB permeability. D-galactose substantially increased Evans blue extravasation, whereas both HY7715 groups showed values close to those of the NOR group. Cortical CLDN5 staining was also reduced after D-galactose exposure, together with lower *Cldn5*, *Tjp1*, and *Ocln* expression. Similar loss of tight junction proteins, structural tight junction abnormalities, impaired antioxidant defenses, and increased BBB permeability have previously been reported in D-galactose-treated C57BL/6 mice [14]. The recovery of CLDN5 staining and tight junction-associated gene expression following HY7715 intake was consistent with the reduction in Evans blue permeability. Glycation-related oxidative and inflammatory stress may link circulating AGE accumulation with BBB impairment, although direct AGE-RAGE signaling was not examined.

The hippocampus is central to learning and memory and is particularly vulnerable to age-related oxidative stress and neuroinflammation. Hippocampal aging involves altered synaptic plasticity and enhanced microglial reactivity, together with inflammatory and metabolic changes [21, 22]. D-galactose increased both the number and soma area of Iba1-positive microglia in the CA1 region and markedly elevated *Tnf-α*, *IL-1β*, and *IL-6*. Both live and heat-killed HY7715 attenuated these microglial morphological and inflammatory changes. These findings are consistent with altered microglial reactivity. However, Iba1-based morphological analysis alone does not fully define the functional activation state or phenotype of microglia. D-galactose also increased *Trp53* (*p53*) and *Cdkn1a* (*p21*), reduced *Gpx1*, *Gpx2*, *Sod1*, and *Sod2*, and shifted apoptosis-related expression toward increased *Bax* and decreased *Bcl-2*. These changes are consistent with the oxidative stress, impaired antioxidant defense, inflammation, and cellular senescence that characterize the D-galactose aging model [10]. Both 7715L and 7715K attenuated these alterations.

These brain results also extend previous findings for HY7715. In a stress-associated neurobiological aging model, HY7715 suppressed hippocampal *p53* and *p21*-related responses, reduced *IL-6*, improved *Gpx1* and *Sod1* expression, and modified the stress-altered gut microbiota [20]. Previous work has also demonstrated biological activity of heat-killed HY7715 and HY7715-derived extracellular vesicles, including effects on mitochondrial and inflammatory responses, while HY7715-derived extracellular vesicles enhanced intestinal tight junction-associated markers [23]. These observations, together with the present results, suggest that bacterial viability may not be essential for several HY7715-associated host responses, although the active components responsible for the effects of 7715K remain unclear.

Given the systemic nature of D-galactose-induced accelerated aging, intestinal homeostasis was also examined. The intestinal tight junction barrier restricts passage of luminal microorganisms and microbial products into underlying tissues and the systemic circulation. Loss of barrier integrity can therefore amplify mucosal and systemic inflammatory signaling [24]. In the CON group, colonic *Tjp1* and *Ocln* were reduced, whereas *Cldn2*, *IL-1β*, *IL-6*, and *Tnf-α* increased. D-galactose-induced intestinal aging has similarly been associated with increased permeability and reduced ZO-1 and occludin expression, together with microbiota alterations [25]. Both HY7715 preparations attenuated the colonic changes. These intestinal improvements occurred in parallel with reduced circulating AGE levels, improved BBB integrity, and reduced neuroinflammation. Although these concurrent changes are consistent with a possible gut–brain axis relationship, the present data do not establish that intestinal or microbial changes mediated the brain-related effects of HY7715.

Given these parallel intestinal changes, we next examined whether D-galactose and HY7715 were associated with alterations in the fecal microbial community. Neither Faith’s PD nor observed features differed significantly among groups, although both indices were numerically higher in CON. The NOR and CON groups also showed no statistically significant difference in unweighted or weighted UniFrac distances, with p values of 0.066 and 0.052, respectively. Accordingly, the data do not indicate a marked community-wide shift between the NOR and CON groups. Instead, several taxon-specific changes were observed. The 7715L group differed significantly from CON in both UniFrac analyses, whereas 7715K did not, showing that viable HY7715 was associated with a detectable difference in overall microbial community structure.

A striking characteristic of the fecal profile was the predominance of *Faecalibaculum*, which accounted for more than 70% of the microbial community across all groups. *Faecalibaculum rodentium* was originally isolated from laboratory mouse feces and produces lactic acid as a major metabolic product [26]. Importantly, gut microbial composition can differ substantially among laboratory C57BL/6 mice according to vendor, breeding facility, housing, environment, and husbandry [27, 28]. Thus, the unusually high abundance observed across all experimental groups may reflect cohort- or facility-associated baseline characteristics rather than a treatment-specific response. *Faecalibaculum* was therefore retained in the original dataset but excluded from a separate visualization to allow clearer evaluation of lower-abundance taxa.

LEfSe analysis identified significantly higher relative abundances of *Desulfovibrio*, *Alistipes*, *Gemella*, and *Anaerotruncus* in the CON group than in the NOR group. *Desulfovibrio* is particularly relevant because naturally aging C57BL/6J mice have shown increased *Desulfovibrio* together with reductions in *Akkermansia* and *Lactobacillus* [29]. Members of *Desulfovibrio* are sulfate-reducing bacteria that produce H_2_S. Experimental administration of *D. vulgaris* has been shown to induce intestinal inflammation and disrupt barrier function in mice [30]. Thus, the increase in *Desulfovibrio* is compatible with the inflammatory colonic phenotype observed in CON, although causality cannot be assigned. *Alistipes* also increased with D-galactose. An age-associated increase in *Alistipes* has previously been reported in mice, supporting its consideration as one of the taxa associated with aging-related microbial changes [31]. Because the biological roles of *Alistipes* vary among species, the present increase should not be interpreted as expansion of a uniformly harmful bacterial group. Both 7715L and 7715K significantly increased *Bifidobacterium* compared with the CON group. Members of this genus are widely associated with intestinal immune homeostasis and regulation of tight junction barrier function [32]. This shared increase may represent one microbiota response common to both forms of HY7715. Live HY7715 produced additional changes that were not apparent after heat-killed HY7715. *Lactiplantibacillus* was significantly increased in the 7715L group compared with the CON group. An ASV showing the highest BLASTn similarity to *L. plantarum* was detected only in the 7715L group, which is consistent with gastrointestinal passage of the administered viable bacteria but does not demonstrate permanent colonization. *Desulfovibrio* was also significantly lower in the 7715L group than in the CON group, consistent with our previous finding that HY7715 reduced this genus in a stress-associated aging model [20]. Whether the decrease resulted from direct competition or an indirectly modified intestinal environment remains unknown. The selective increase in an ASV putatively assigned to *B. pseudolongum* in the 7715L group is also of interest. Previous co-culture studies using *L. plantarum* and *Bifidobacterium* species have demonstrated metabolic interactions between these bacteria [33, 34]. Although these studies did not examine HY7715 or *B. pseudolongum* directly, a similar ecological interaction may have contributed to the increase observed following live HY7715 intake. Specific *B. pseudolongum* strains have also been reported to improve intestinal tight junction-associated proteins and inflammatory responses in experimental colitis [35]. The selective increase in the *B. pseudolongum*-assigned ASV therefore provides an interesting microbial feature of the 7715L group, although the underlying interaction requires direct experimental confirmation.

The correlation analysis was used as supportive rather than mechanistic evidence. *Bifidobacterium* was negatively correlated with serum AGEs and hippocampal Iba1-positive soma area and positively correlated with cortical CLDN5-positive area. *Akkermansia* showed a negative correlation with Evans blue leakage, whereas *Desulfovibrio* showed a positive correlation. Because Spearman correlation does not establish temporal sequence or causality, these results indicate associations between microbial taxa and AGE-, BBB-, and neuroinflammation-related markers rather than direct microbial regulation of these phenotypes.

Some limitations should be acknowledged. The concurrent changes in AGE accumulation, intestinal barrier-related markers, BBB permeability, neuroinflammation, and microbiota composition were observed within the same experimental framework, but the relationships among these responses remain to be clarified. Species-level assignments based on 16S rRNA sequencing and BLASTn remain putative, and fecal detection of an ASV assigned to *L. plantarum* does not distinguish transient passage from stable colonization. In addition, the D-galactose model captures selected features of accelerated aging rather than the full complexity of natural aging. Only male mice were included in the present study; therefore, potential sex-dependent differences were not assessed, and future studies including both sexes are warranted.

In conclusion, both live and heat-killed HY7715 attenuated several aging-related alterations induced by D-galactose, including increased circulating AGE levels, BBB dysfunction, neuroinflammation, altered antioxidant- and apoptosis-related responses, and colonic barrier-associated changes. Although the two preparations showed generally similar effects on these aging-related host responses, they were associated with changes in selected gut microbial taxa, while a significant difference in overall microbial community structure relative to CON was observed only in the 7715L group. These findings suggest that both live and heat-killed HY7715 attenuate systemic, intestinal, and neurobiological alterations associated with accelerated aging, accompanied by changes in gut microbiota composition.

## Supplemental Materials

Supplementary data for this paper are available on-line only at http://jmb.or.kr.



## Figures and Tables

**Fig. 1:**
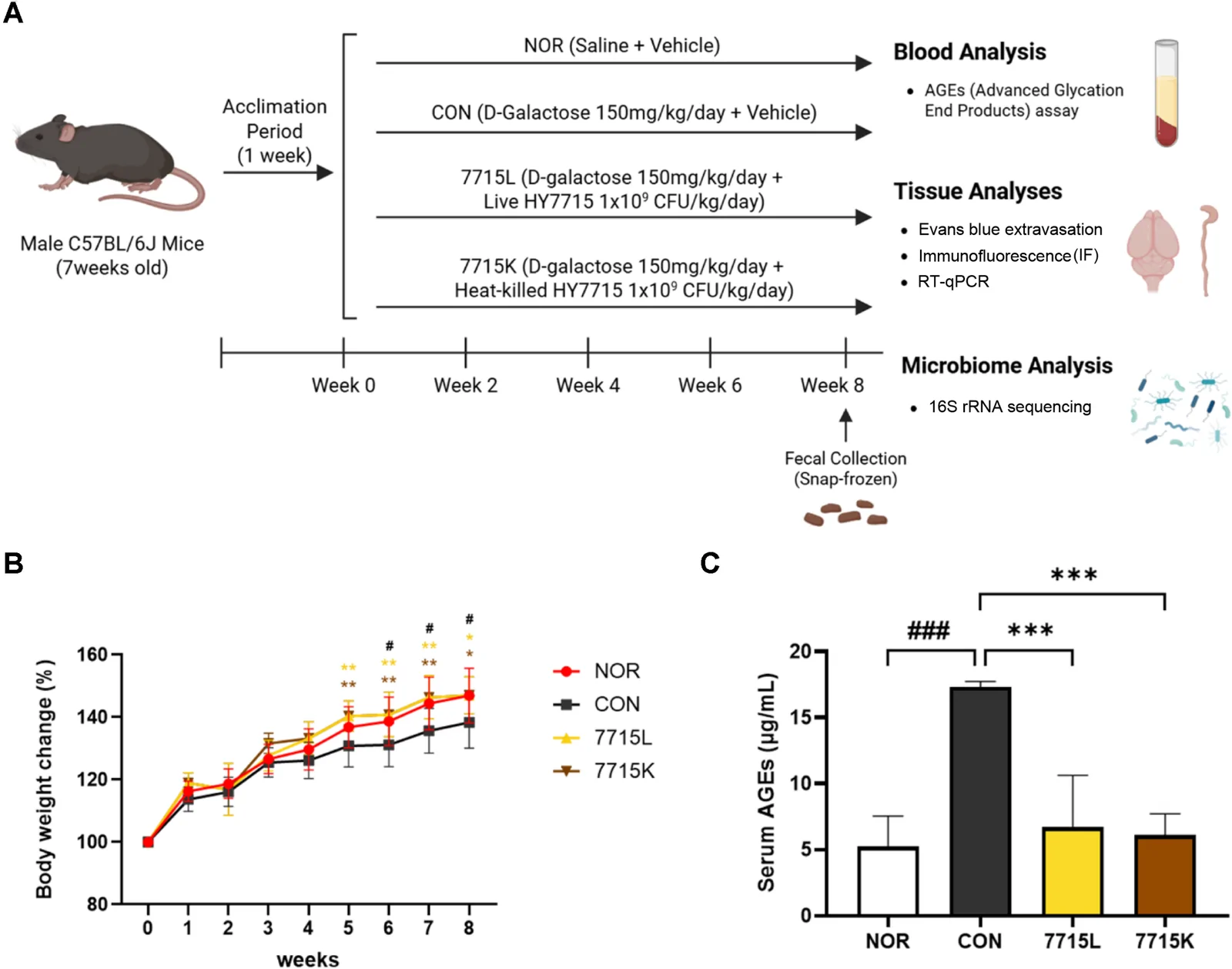
Experimental design and effects of HY7715 supplementation on body weight change and circulating advanced glycation end products in D-galactose-induced accelerated aging mice. (**A**) Illustration of the experimental design of the D-galactose-induced accelerated aging model. (**B**) Body weight change was monitored weekly for 8 weeks and expressed as percentage change from week 0. (**C**) Quantification of serum advanced glycation end products (AGEs) levels (μg/mL). NOR, untreated normal group. CON, D-galactose-induced control group. 7715L, D-galactose-induced mice administered live HY7715 at 1 × 10^9^ CFU/kg/day. 7715K, D-galactose-induced mice administered heat-killed HY7715 equivalent to 1 × 10^9^ CFU/kg/day before heat killing. Data are presented as mean ± SD. Statistical significance was determined by two-way ANOVA followed by Dunnett’s multiple comparisons test for body weight changes and by one-way ANOVA followed by Dunnett’s multiple comparisons test for other analyses. *, **, and *** indicate *p* < 0.05, *p* < 0.01, and *p* < 0.001 compared with CON, respectively. #, ##, and ### indicate *p* < 0.05, *p* < 0.01, and *p* < 0.001 compared with NOR, respectively.

**Fig. 2:**
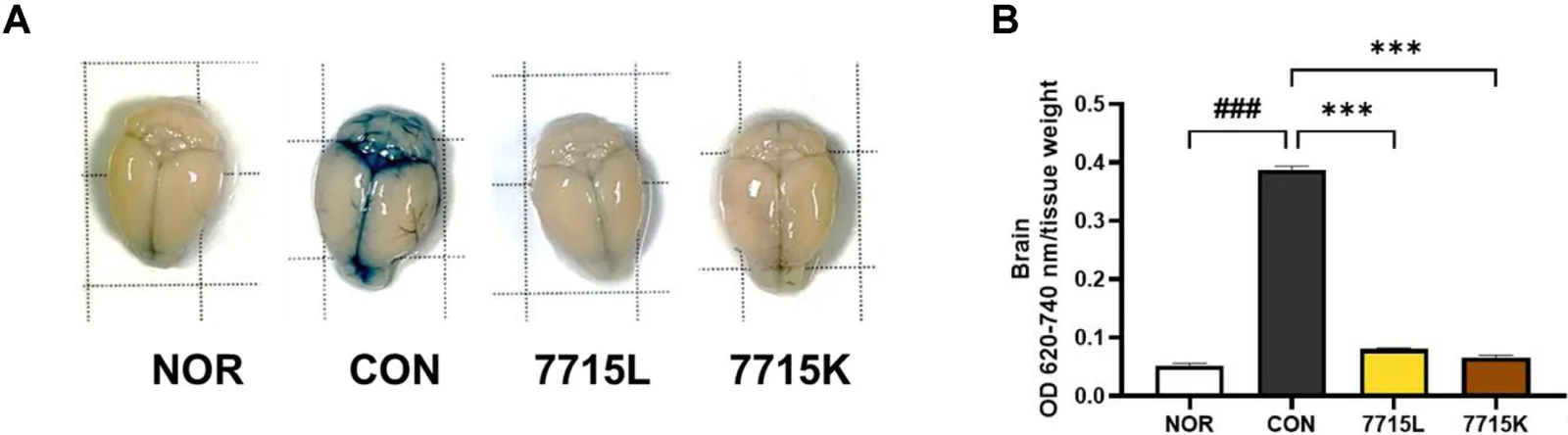
Effects of HY7715 supplementation on blood-brain barrier permeability in D-galactose-induced accelerated aging mice. (**A**) Representative images of brain tissues showing Evans blue extravasation. (**B**) Quantification of Evans blue extravasation in brain tissue, expressed as absorbance normalized to tissue weight. NOR, untreated normal group. CON, D-galactose-induced control group. 7715L, D-galactose-induced mice administered live HY7715 at 1 × 10^9^ CFU/kg/day. 7715K, D-galactose-induced mice administered heat-killed HY7715 equivalent to 1 × 10^9^ CFU/kg/day before heat killing. Data are presented as mean ± SD. Statistical significance was determined by one-way ANOVA followed by Dunnett’s multiple comparisons test. Statistical symbols indicate selected comparisons between NOR and CON, and between CON and HY7715-treated groups. #, ##, and ### indicate *p* < 0.05, *p* < 0.01, and *p* < 0.001 compared with NOR, respectively. *, **, and *** indicate *p* < 0.05, *p* < 0.01, and *p* < 0.001 compared with CON, respectively.

**Fig. 3:**
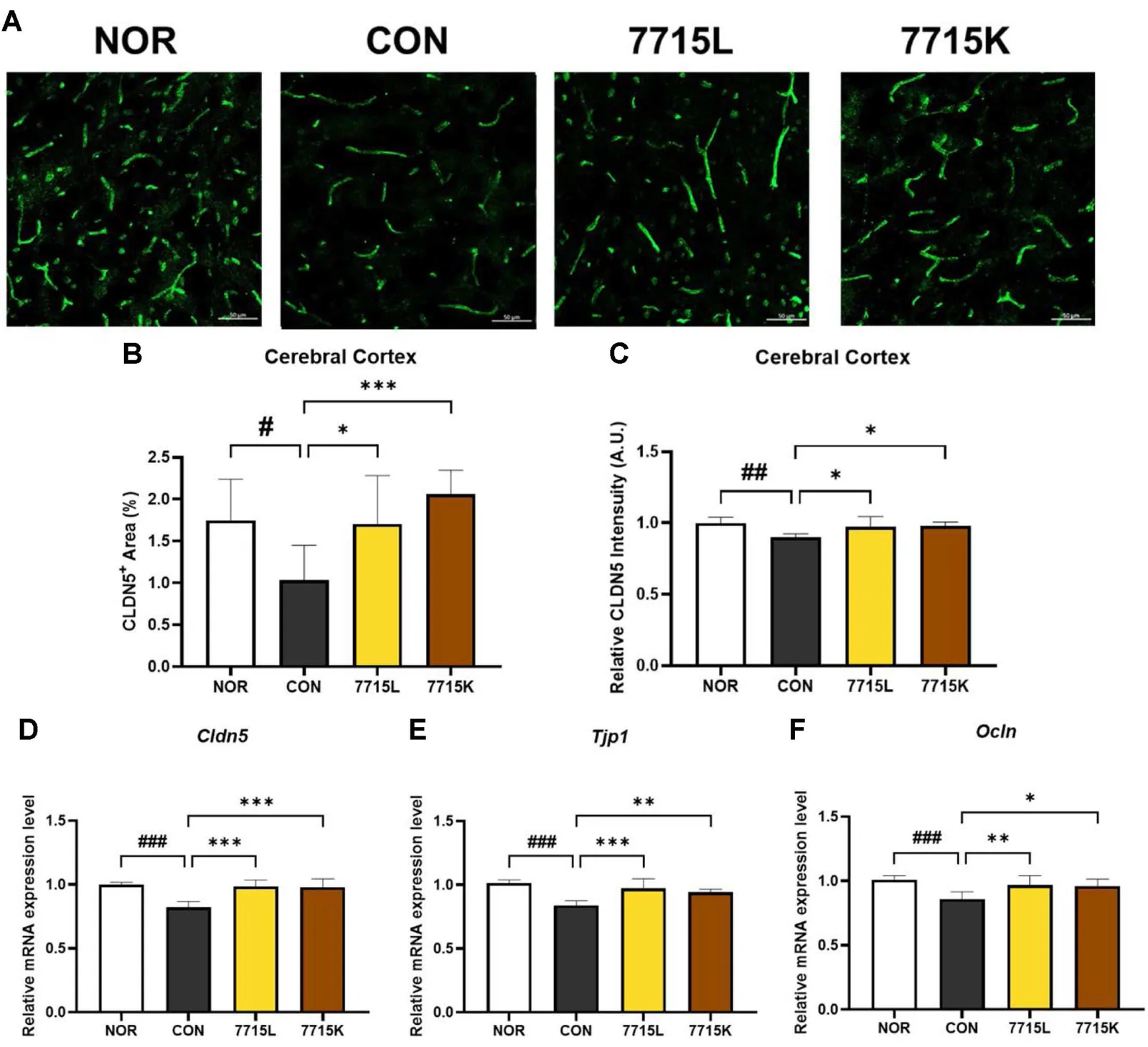
Effects of HY7715 supplementation on cortical tight junction-associated markers in D-galactose-induced accelerated aging mice. (**A**) Representative immunofluorescence images of CLDN5 in the cerebral cortex. (**B**) Quantification of CLDN5-positive area in the cerebral cortex. (**C**) Relative fluorescence intensity of CLDN5 in the cerebral cortex. Relative mRNA expression levels in the cerebral cortex were determined by RT-qPCR, normalized to GAPDH, and expressed as fold changes relative to the NOR group. (**D**) *Cldn5*, (**E**) *Tjp1*, (**F**) *Ocln*. NOR, untreated normal group. CON, D-galactose-induced control group. 7715L, D-galactose-induced mice administered live HY7715 at 1 × 10^9^ CFU/kg/day. 7715K, D-galactose-induced mice administered heat-killed HY7715 equivalent to 1 × 10^9^ CFU/kg/day before heat killing. Data are presented as mean ± SD. Statistical significance was determined by one-way ANOVA followed by Dunnett’s multiple comparisons test. Statistical symbols indicate selected comparisons between NOR and CON, and between CON and HY7715-treated groups. #, ##, and ### indicate *p* < 0.05, *p* < 0.01, and *p* < 0.001 compared with NOR, respectively. *, **, and *** indicate *p* < 0.05, *p* < 0.01, and *p* < 0.001 compared with CON, respectively.

**Fig. 4:**
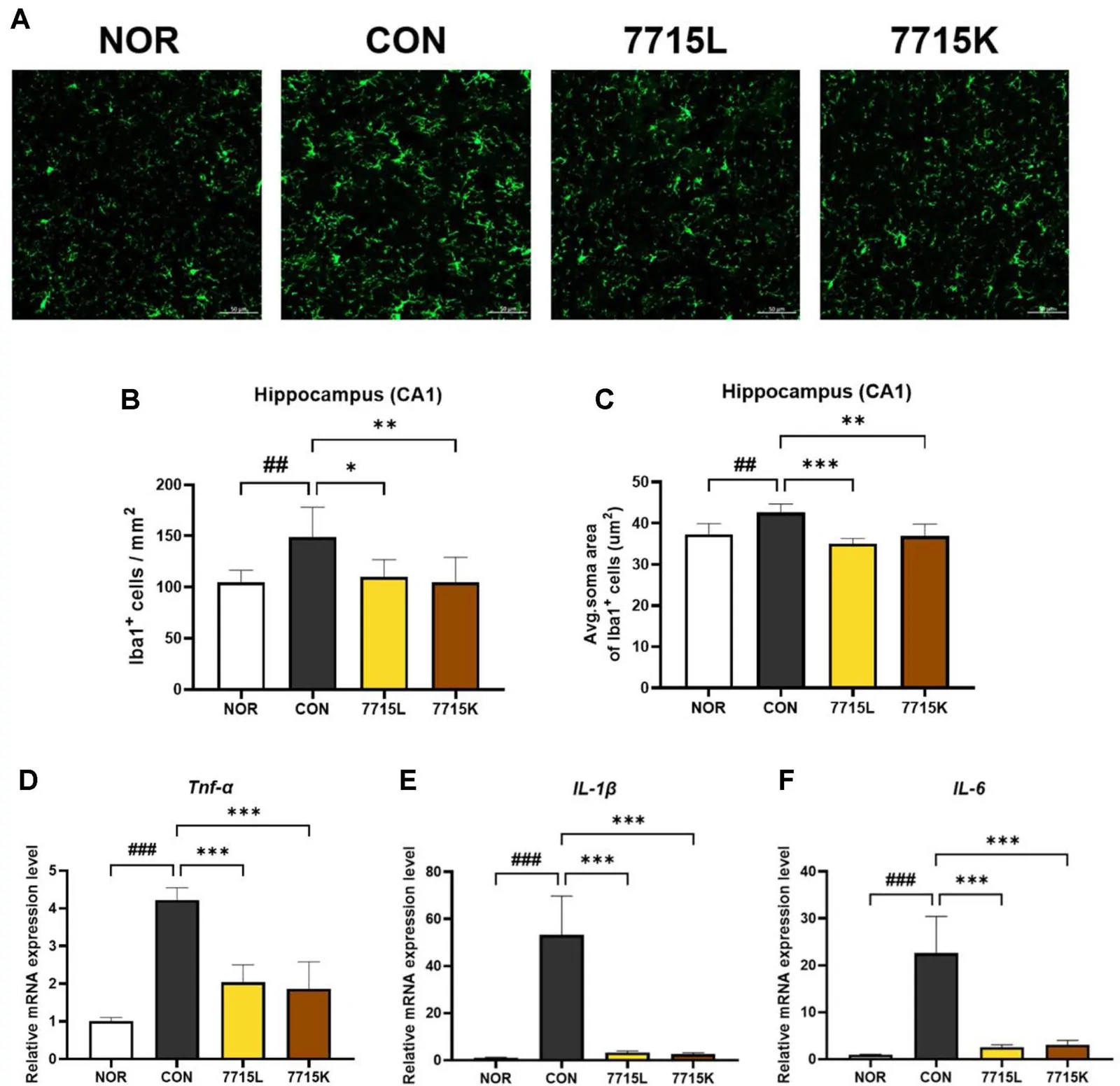
Effects of HY7715 supplementation on hippocampal microglial reactivity and inflammatory gene expression in D-galactose-induced accelerated aging mice. (**A**) Representative immunofluorescence images of Iba1 in the hippocampal CA1 region. (**B**) Quantification of Iba1-positive cells in the hippocampal CA1 region. (**C**) Average soma area of Iba1-positive cells in the hippocampal CA1 region. Relative mRNA expression levels in the hippocampus were determined by RT-qPCR, normalized to GAPDH, and expressed as fold changes relative to the NOR group. (**D**) *Tnf-α*, (**E**) *IL-1β*, (**F**) *IL-6*. NOR, untreated normal group. CON, D-galactose-induced control group. 7715L, D-galactose-induced mice administered live HY7715 at 1 × 10^9^ CFU/kg/day. 7715K, D-galactose-induced mice administered heat-killed HY7715 equivalent to 1 × 10^9^ CFU/kg/day before heat killing. Data are presented as mean ± SD. Statistical significance was determined by one-way ANOVA followed by Dunnett’s multiple comparisons test. Statistical symbols indicate selected comparisons between NOR and CON, and between CON and HY7715-treated groups. #, ##, and ### indicate *p* < 0.05, *p* < 0.01, and *p* < 0.001 compared with NOR, respectively. *, **, and *** indicate *p* < 0.05, *p* < 0.01, and *p* < 0.001 compared with CON, respectively.

**Fig. 5:**
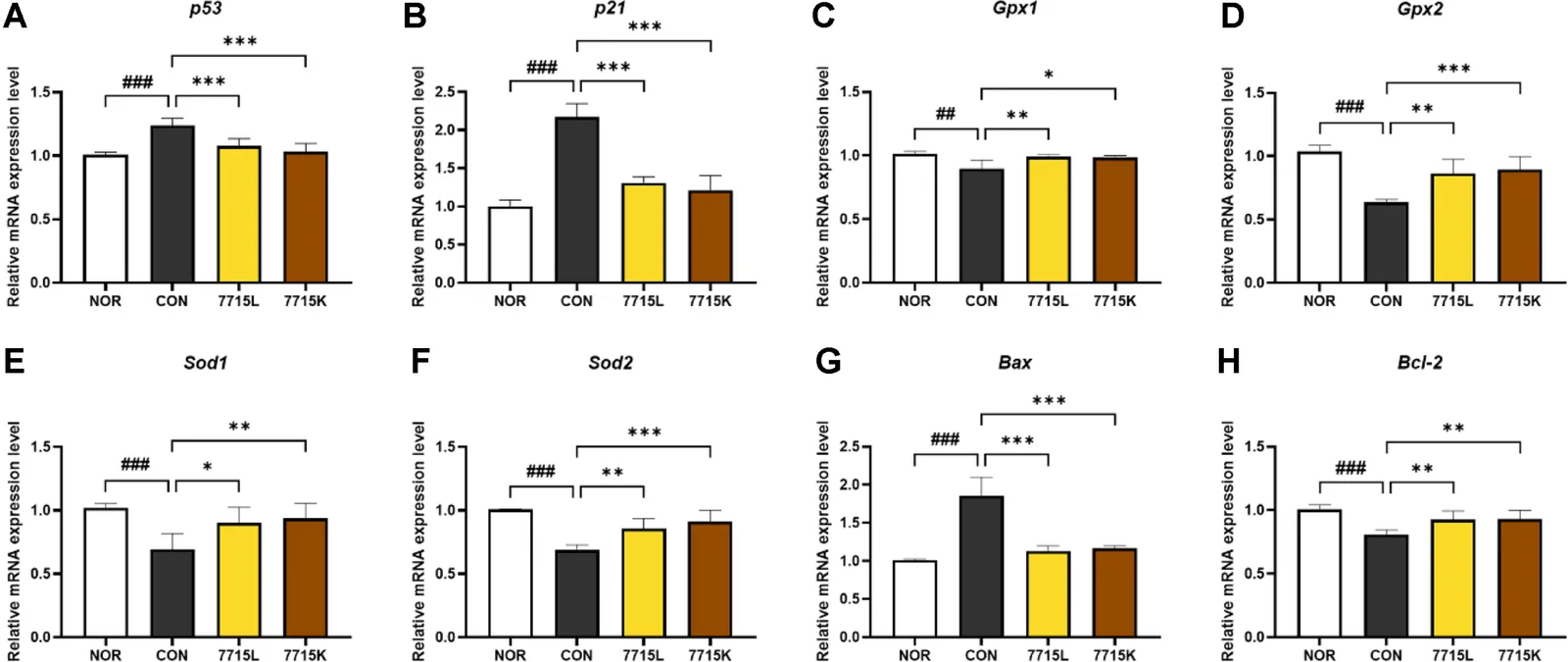
Effects of HY7715 supplementation on senescence-, antioxidant-, and apoptosis-related gene expression in the hippocampus of D-galactose-induced accelerated aging mice. Relative mRNA expression levels in the hippocampus were determined by RT-qPCR, normalized to GAPDH, and expressed as fold changes relative to the NOR group. (**A**) *p53*, (**B**) *p21*, (**C**) *Gpx1*, (**D**) *Gpx2*, (**E**) *Sod1*, (**F**) *Sod2*, (**G**) *Bax*, (**H**) *Bcl-2*. NOR, untreated normal group. CON, D-galactose-induced control group. 7715L, D-galactose-induced mice administered live HY7715 at 1 × 10^9^ CFU/kg/day. 7715K, D-galactose-induced mice administered heat-killed HY7715 equivalent to 1 × 10^9^ CFU/kg/day before heat killing. Data are presented as mean ± SD. Statistical significance was determined by one-way ANOVA followed by Dunnett’s multiple comparisons test. *, **, and *** indicate *p* < 0.05, *p* < 0.01, and *p* < 0.001 compared with CON, respectively. #, ##, and ### indicate *p* < 0.05, *p* < 0.01, and *p* < 0.001 compared with NOR, respectively.

**Fig. 6:**
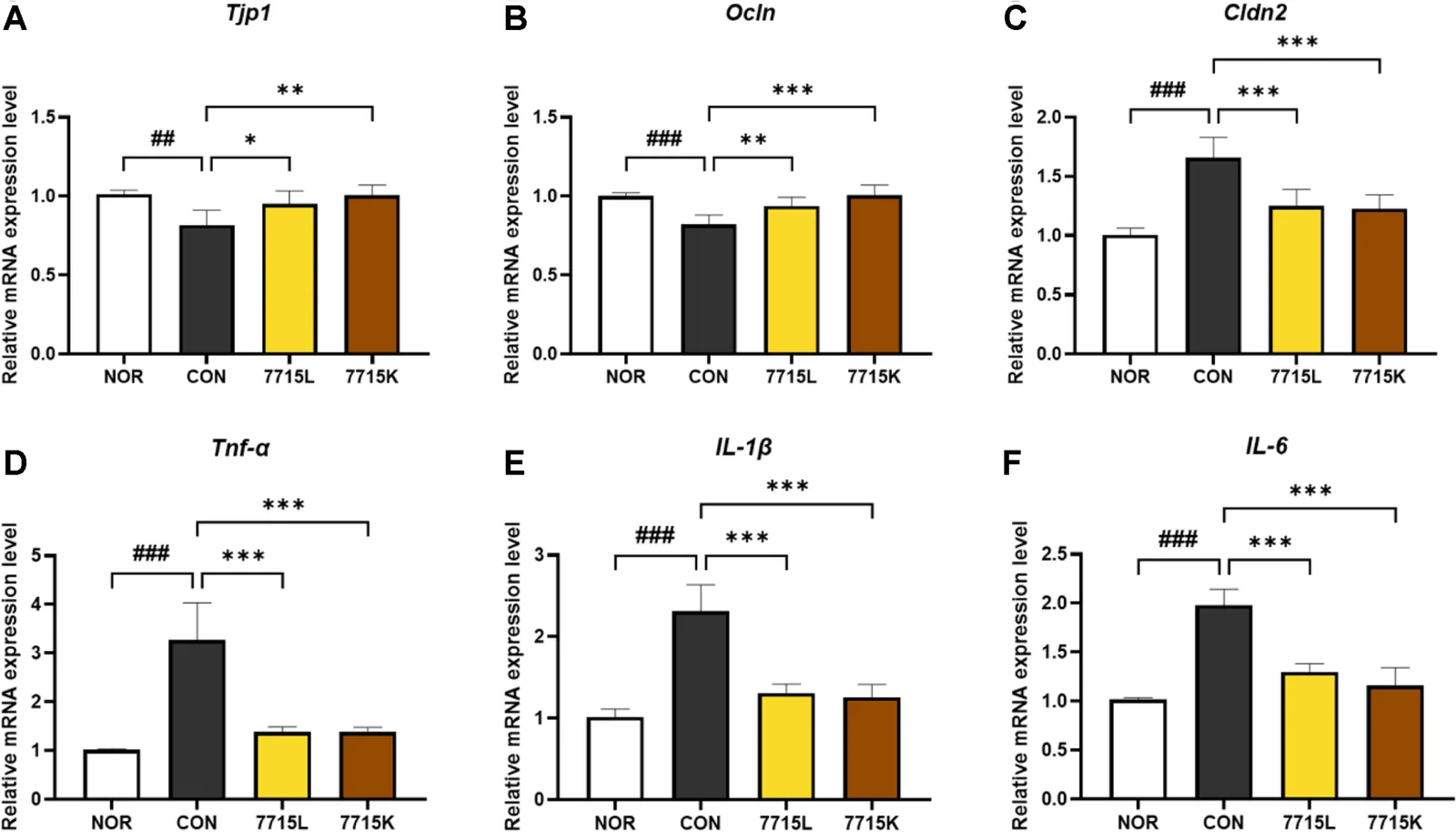
Effects of HY7715 supplementation on colonic tight junction and inflammation-related gene expression in D-galactose-induced accelerated aging mice. Relative mRNA expression levels in the colon were determined by RT-qPCR, normalized to GAPDH, and expressed as fold changes relative to the NOR group. (**A**) *Tjp1*, (**B**) *Ocln*, (**C**) *Cldn2*, (**D**) *Tnf-α*, (**E**) *IL-1β*, (**F**) *IL-6*. NOR, untreated normal group. CON, D-galactose-induced control group. 7715L, D-galactose-induced mice administered live HY7715 at 1 × 10^9^ CFU/kg/day. 7715K, D-galactose-induced mice administered heat-killed HY7715 equivalent to 1 × 10^9^ CFU/kg/day before heat killing. Data are presented as mean ± SD. Statistical significance was determined by one-way ANOVA followed by Dunnett’s multiple comparisons test. *, **, and *** indicate *p* < 0.05, *p* < 0.01, and *p* < 0.001 compared with CON, respectively. #, ##, and ### indicate *p* < 0.05, *p* < 0.01, and *p* < 0.001 compared with NOR, respectively.

**Fig. 7:**
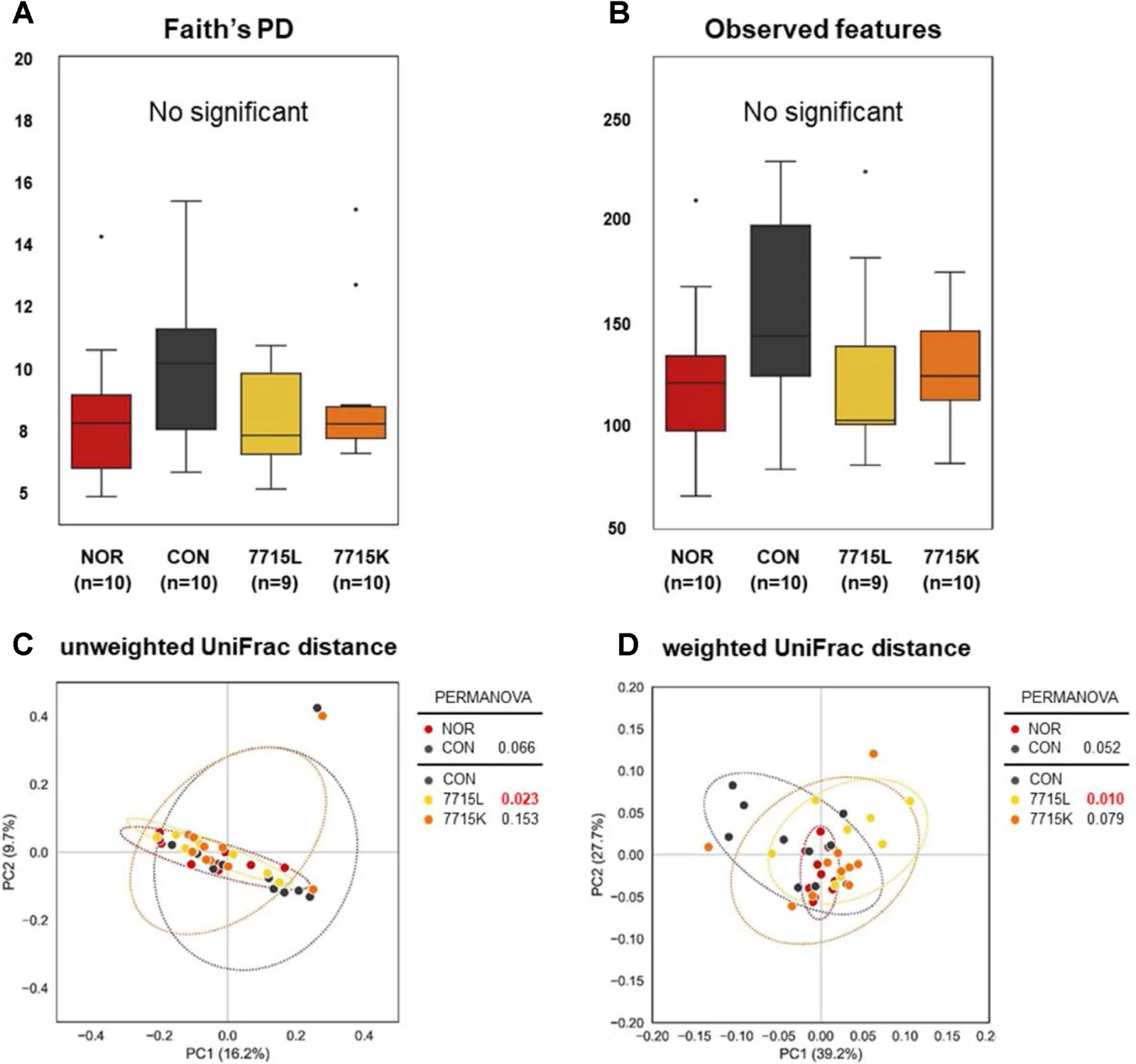
Effects of HY7715 supplementation on gut microbial alpha and beta diversity in D-galactose-induced accelerated aging mice. Fecal microbiota composition was analyzed by 16S rRNA amplicon sequencing. (**A**) Faith’s phylogenetic diversity (PD). (**B**) Observed features. (**C**) Principal coordinate analysis based on unweighted UniFrac distance. (**D**) Principal coordinate analysis based on weighted UniFrac distance. NOR, untreated normal group. CON, D-galactose-induced control group. 7715L, D-galactose-induced mice administered live HY7715 at 1 × 10^9^ CFU/kg/day. 7715K, D-galactose-induced mice administered heat-killed HY7715 equivalent to 1 × 10^9^ CFU/kg/day before heat killing. Alpha diversity was analyzed using the Kruskal-Wallis test. Beta diversity was visualized by principal coordinate analysis and differences among groups were assessed by PERMANOVA.

**Fig. 8:**
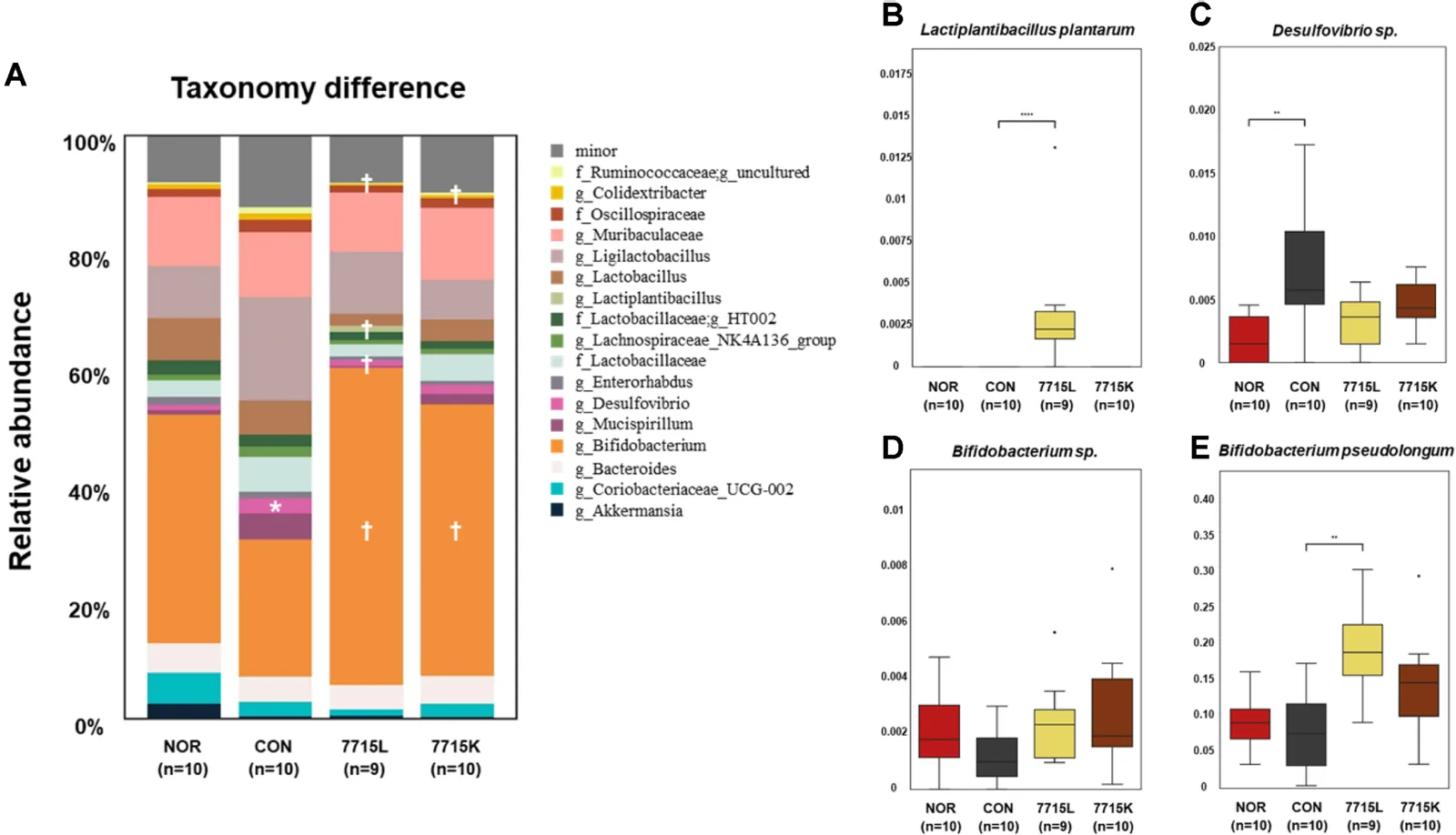
Effects of HY7715 supplementation on fecal microbial taxonomic composition in D-galactose-induced accelerated aging mice. (**A**) Relative abundance of fecal bacterial taxa after excluding the dominant genus *Faecalibaculum*. The remaining taxa were re-normalized to 100% to visualize changes in low-abundance taxa. Taxa with a relative abundance of 1% or higher in at least one group were classified as major taxa, whereas taxa below 1% were grouped as Minor. Differentially abundant taxa were identified by LEfSe analysis with an LDA score > 2.0. Asterisks indicate taxa significantly differing between NOR and CON. Dagger symbols indicate taxa significantly differing between CON and HY7715-treated groups. (**B-E**) Species-level inference of selected bacterial taxa based on BLASTn analysis using the NCBI database. (**B**) *Lactiplantibacillus plantarum*. (**C**) *Desulfovibrio* sp. (**D**) *Bifidobacterium* sp. (**E**) *Bifidobacterium pseudolongum*. NOR, untreated normal group. CON, D-galactose-induced control group. 7715L, D-galactose-induced mice administered live HY7715. 7715K, D-galactose-induced mice administered heat-killed HY7715.

**Fig. 9:**
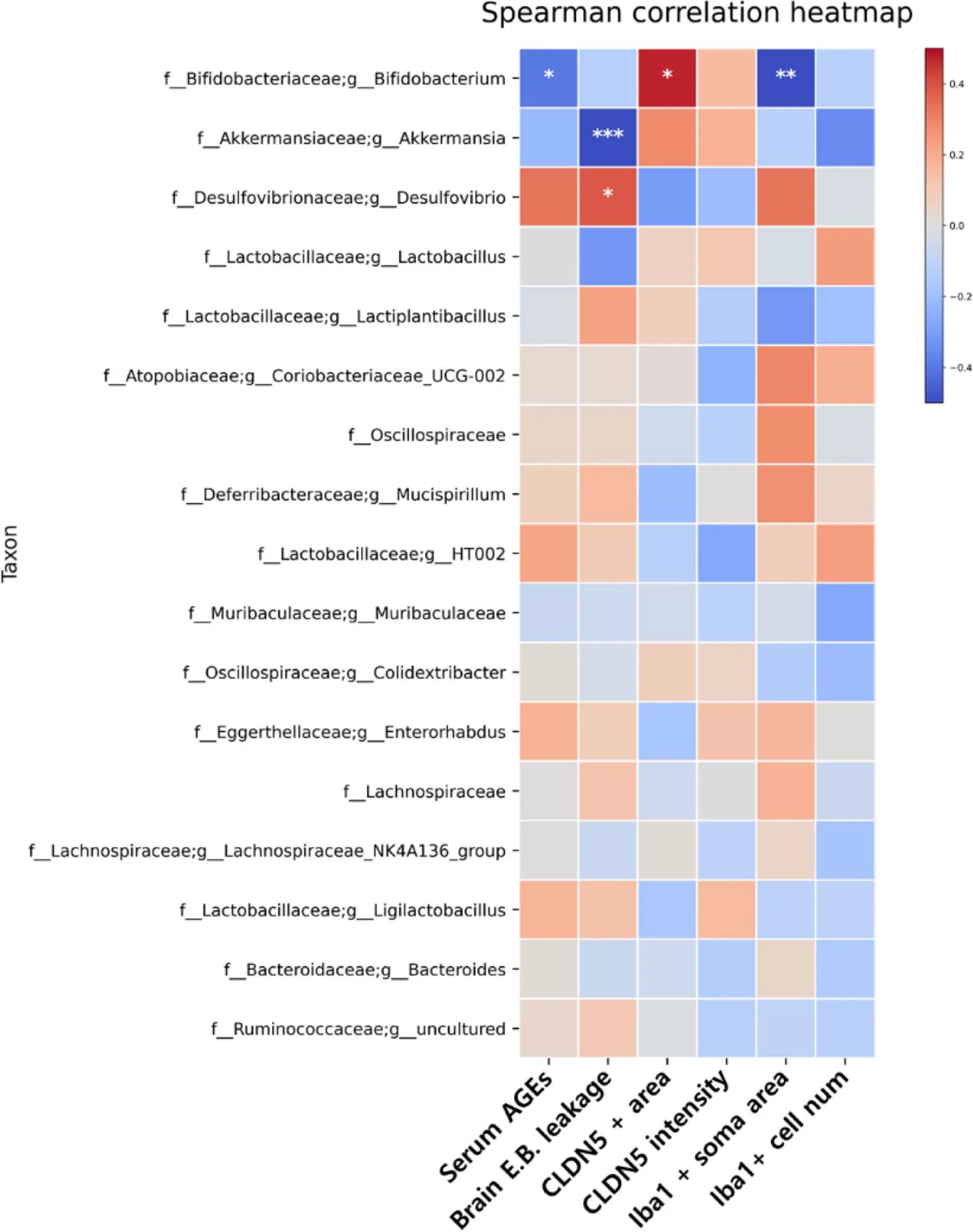
Correlations between fecal microbial taxa and aging-related phenotypic markers in D-galactose-induced accelerated aging mice. Spearman correlation heatmap showing associations between differentially abundant fecal bacterial taxa and aging-related phenotypic markers, including serum AGEs, brain Evans blue leakage, cortical CLDN5-positive area, cortical CLDN5 fluorescence intensity, hippocampal Iba1-positive soma area, and hippocampal Iba1-positive cell number. Asterisks indicate significant correlations, *p* < 0.05. NOR, untreated normal group. CON, D-galactose-induced control group. 7715L, D-galactose-induced mice administered live HY7715. 7715K, D-galactose-induced mice administered heat-killed HY7715.

**Table 1:** TaqMan Gene Expression Assays Used in This Study.

Target gene	Gene symbol	TaqMan Assay ID
Claudin-5	*Cldn5*	Mm00727012_s1
Tight junction protein 1	*Tjp1*	Mm00493699_m1
Occludin	*Ocln*	Mm00500910_m1
Tumor necrosis factor	*Tnf-α*	Mm00443258_m1
Interleukin-1 beta	*Il1b*	Mm00434228_m1
Interleukin-6	*Il6*	Mm00446190_m1
Tumor protein *p53*	*Trp53 (p53)*	Mm01731290_g1
Cyclin-dependent kinase inhibitor 1A	*Cdkn1a (p21)*	Mm04205640_g1
Glutathione peroxidase 1	*Gpx1*	Mm04207457_g1
Glutathione peroxidase 2	*Gpx2*	Mm01286848_gH
Superoxide dismutase 1	*Sod1*	Mm01344233_g1
Superoxide dismutase 2	*Sod2*	Mm01313000_m1
BCL2-associated X protein	*Bax*	Mm00432051_m1
B-cell lymphoma 2	*Bcl2*	Mm00477631_m1
Claudin-2	*Cldn2*	Mm00516703_s1
Glyceraldehyde-3-phosphate dehydrogenase	*Gapdh*	Mm99999915_g1
