# Supplementary material for: Live and Heat-Killed Lactiplantibacillus plantarum HY7715 Attenuate D-Galactose-Induced Aging-Related Brain Changes and Blood-Brain Barrier Disruption with Gut Microbiota Modulation in Mice
Source: J Microbiol Biotechnol. 2026 Oct 21;36:e2609057. doi: 10.4014/jmb.2609.09057 (PMC13687644; doi:10.4014/jmb.2609.09057)
Supplement: Supplementary file 1 [file jmb-36-e2609057-supple.pdf]

J. Microbiol. Biotechnol. 2026. 36: e2609057
https://doi.org/10.4014/jmb.2609.09057

Supplementary Figures

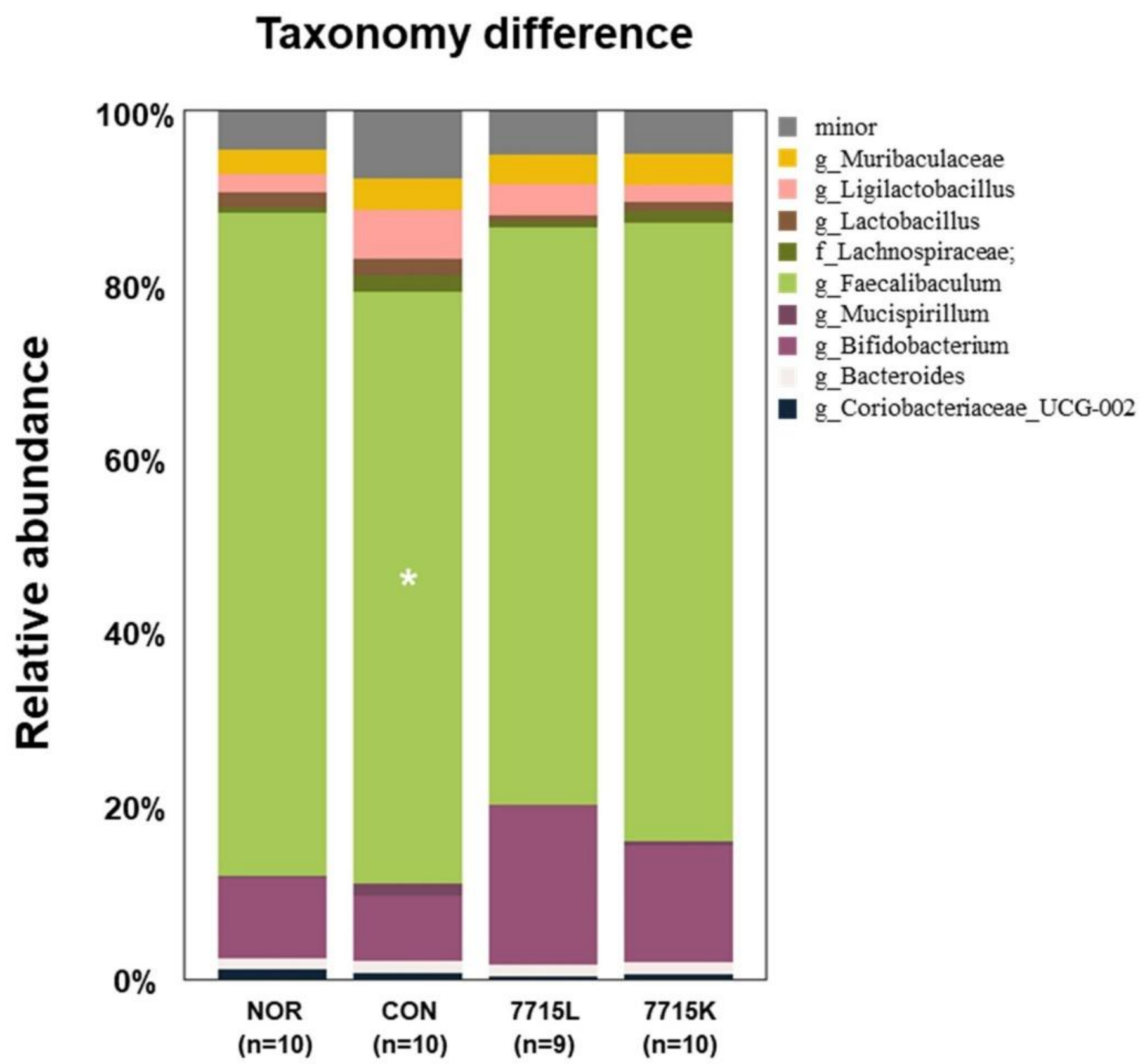

Fig. S1. **Fecal microbiota composition was analyzed by 16S rRNA amplicon sequencing and presented as relative abundance**. Taxa with a relative abundance of 1% or higher in at least one group were classified as major taxa, whereas taxa below 1% were grouped as Minor. Differentially abundant taxa were identified by LEfSe analysis with an LDA score > 2.0. Asterisk indicates taxa differing between NOR and CON, and between CON and HY7715-treated groups. NOR, untreated normal group. CON, D-galactose-induced control group. 7715L, D-galactose-induced mice administered live HY7715. 7715K, D-galactose-induced mice administered heat-killed HY7715.

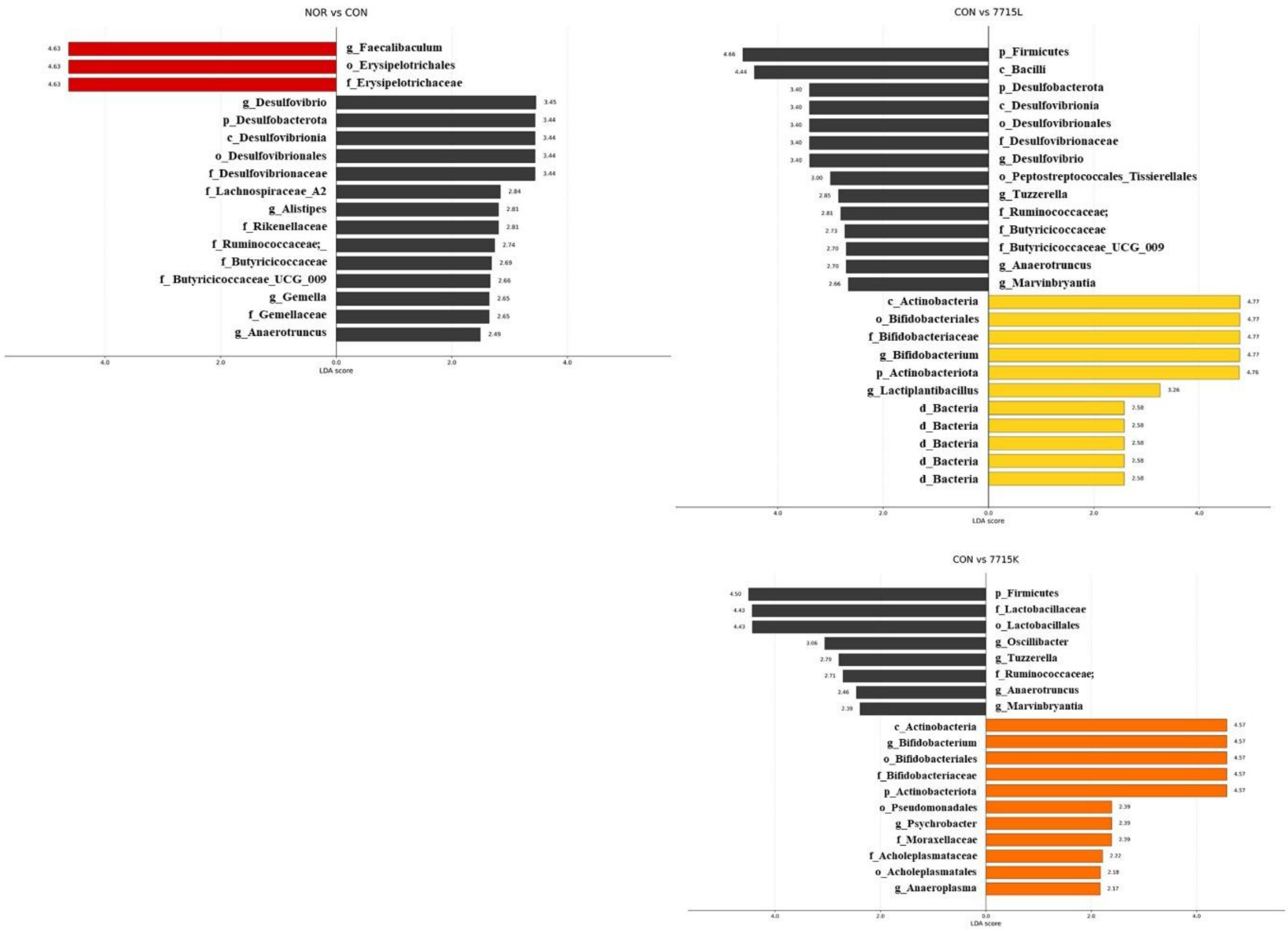

Fig. S2. **LEfSe analysis of fecal microbial taxa among experimental groups.** Differentially abundant taxa were identified using LEfSe based on level 6 taxonomic profiles. Pairwise comparisons are shown for NOR versus CON, CON versus 7715L, and CON versus 7715K. Taxa with an LDA score > 2.0 are shown. NOR, untreated normal group. CON, D-galactose-induced control group. 7715L, D-galactose-induced mice administered live HY7715. 7715K, D-galactose-induced mice administered heat-killed HY7715.
